# Integrating genomic and infrared spectral data improves the prediction of milk protein composition in dairy cattle

**DOI:** 10.1186/s12711-021-00620-7

**Published:** 2021-03-16

**Authors:** Toshimi Baba, Sara Pegolo, Lucio F. M. Mota, Francisco Peñagaricano, Giovanni Bittante, Alessio Cecchinato, Gota Morota

**Affiliations:** 1 Department of Animal and Poultry Sciences, Virginia Polytechnic Institute and State University, Blacksburg, VA 24061 USA; 2 Department of Agronomy, Food, Natural Resources, Animals and Environment (DAFNAE), University of Padova, Viale dell’Università 16, 35020 Legnaro, Italy; 3 Department of Animal and Dairy Sciences, University of Wisconsin-Madison, Madison, WI 53706 USA; 4 Center for Advanced Innovation in Agriculture, Virginia Polytechnic Institute and State University, Blacksburg, VA 24061 USA

## Abstract

**Background:**

Over the past decade, Fourier transform infrared (FTIR) spectroscopy has been used to predict novel milk protein phenotypes. Genomic data might help predict these phenotypes when integrated with milk FTIR spectra. The objective of this study was to investigate prediction accuracy for milk protein phenotypes when heterogeneous on-farm, genomic, and pedigree data were integrated with the spectra. To this end, we used the records of 966 Italian Brown Swiss cows with milk FTIR spectra, on-farm information, medium-density genetic markers, and pedigree data. True and total whey protein, and five casein, and two whey protein traits were analyzed. Multiple kernel learning constructed from spectral and genomic (pedigree) relationship matrices and multilayer BayesB assigning separate priors for FTIR and markers were benchmarked against a baseline partial least squares (PLS) regression. Seven combinations of covariates were considered, and their predictive abilities were evaluated by repeated random sub-sampling and herd cross-validations (CV).

**Results:**

Addition of the on-farm effects such as herd, days in milk, and parity to spectral data improved predictions as compared to those obtained using the spectra alone. Integrating genomics and/or the top three markers with a large effect further enhanced the predictions. Pedigree data also improved prediction, but to a lesser extent than genomic data. Multiple kernel learning and multilayer BayesB increased predictive performance, whereas PLS did not. Overall, multilayer BayesB provided better predictions than multiple kernel learning, and lower prediction performance was observed in herd CV compared to repeated random sub-sampling CV.

**Conclusions:**

Integration of genomic information with milk FTIR spectral can enhance milk protein trait predictions by 25% and 7% on average for repeated random sub-sampling and herd CV, respectively. Multiple kernel learning and multilayer BayesB outperformed PLS when used to integrate heterogeneous data for phenotypic predictions.

## Background

Large-scale phenotyping is critical for efficient farm management and successful breeding programs [1]. Fourier transform infrared (FTIR) spectroscopy is a low-cost, non-destructive, and rapid technology that scans milk samples with an infrared light to detect specific chemical bonds [2]. FTIR spectroscopy has been routinely used to predict the chemical composition of milk in most herd recording programs [3, 4]. Recent studies have used milk FTIR spectral information to develop equations for predicting complex traits that are difficult and expensive to measure because of high phenotyping costs. These include milk fatty acids [5], energy intake [6], methane emissions [7], and metabolic profiles [8]. Milk FTIR spectral bands have also been used to predict cow health and pregnancy [9–12]. Milk protein composition, especially the casein component, is associated with cheese making, an important trait for the dairy industry [13–15], and thus recent studies have attempted to leverage milk FTIR spectra for large-scale phenotyping for genetic selection [16]. Milk FTIR spectra can also be used for genetic improvement when there are strong additive genetic correlations between target traits and FTIR predictions [17, 18].

Recent technological advancements in phenotyping such as precision agriculture or high-throughput phenotyping create an opportunity to integrate multiple sources of information into a single statistical framework [1]. These sources may capture various signals affecting phenotypes and thus could be combined to improve prediction performance. Enhanced prediction performance of dairy cow fertility was reported for a model integrating milk spectra and on-farm data including herd, days in milk (DIM), and parity [10, 11]. The inclusion of genotype information slightly increased prediction accuracy further [11]. Wang and Bovenhuis [19] stated that combining milk FTIR spectra and polymorphisms located in known genes enhances the prediction accuracy of milk fat composition. Therefore, integrating milk FTIR spectra with genomic data may constitute an alternative strategy for improving prediction accuracy.

Partial least squares (PLS) regression is frequently used in spectral analysis [20]. However, it does not permit different weights or priors to be directly assigned to each source of information in a straightforward manner, particularly when spectral and genomic data are integrated. Here, we hypothesized that a model handling heterogeneous data sources, including on-farm variables, milk FTIR spectra, genomic data, and pedigree information, can enhance prediction, especially when genomic or pedigree data capture phenotypic variation that milk spectra do not explain. The objective of this study was to assess the prediction of milk protein composition by integrating milk FTIR spectra, on-farm data, and genomic or pedigree information with cross-validation (CV). The second objective was to compare the predictive performance of two alternative statistical models. We evaluated multiple kernel learning coupled with spectral and genomic (pedigree) relationship matrices constructed from the spectral and genomic (pedigree) profiles of individuals, respectively. We also assessed multilayer Bayesian variable selection by setting separate mixture priors on the spectral and genetic terms. Then, we compared the prediction performance of these two methods with that of PLS.

## Methods

### Data

In total, 966 Italian Brown Swiss cows with phenotypes, spectra, and genotypes were used in this study. Milk samples were collected from 85 commercial herds in Trento, Italy. More details about data collection are given in [5]. The average cow DIM and parity were 169.8 ± 101.8 and 2.4 ± 1.2, respectively. The average number of cows per herd was 11.4 ± 2.4. Two milk samples of each cow were collected and immediately refrigerated at 4 °C. One sample was transported to the milk quality laboratory at the Trento Breeders Federation (Trento, Italy) for milk composition analysis. The other sample was used for the milk protein analysis by a validated reversed-phase high-performance liquid chromatography (RP-HPLC) method [21].

The following traits were measured: true protein nitrogen (TP), total casein (TCN), total whey protein (TWP), $\kappa \text {-}$CN, $\beta \text {-}$CN, $\alpha _{S1}\text {-}$CN, $\alpha _{S2}\text {-}$CN casein fractions, and $\beta \text {-}$lactoglobulin ($\beta \text {-}$LG) and $\alpha \text {-}$lactalbumin ($\alpha \text {-}$LA) whey proteins. Here, TP comprises TCN and TWP, and is obtained by subtracting non-protein nitrogen (N) from total nitrogen. The fraction traits were calculated as % of total milk nitrogen content. The traits were then summed and subtracted from the total N content of the milk [22]. Milk FTIR spectral data included 1060 wavenumbers in the range of 5011 to 925 (cm$^{-1}$) for each cow determined with a MilkoScan FT6000 (Foss, Hillerød, Denmark). Two spectral acquisitions were obtained for each milk sample and averaged before analysis. Pre-treatment of milk spectra was performed by checking the Mahalanobis distance after conducting principal component analysis. This analysis suggested four animals as potential outliers. However, the removal of these individuals did not influence predictive performance, and hence, we used the data without the pre-treatment.

All the cows in this study were genotyped with an Illumina BovineSNP50 v.2 BeadChip (Illumina, San Diego, CA, USA). Missing genotypes were imputed using a binomial distribution based on the frequency of the reference allele. After removing single nucleotide polymorphisms (SNPs) call rates $< 0.95$ and minor allele frequencies $< 0.05$, 37,519 SNPs were retained for subsequent analyses. Table 1 presents the descriptive statistics of milk protein composition.

**Table 1:** Descriptive statistics of 966 cows for milk protein related phenotypes

Traits^a^	Mean	SD
True protein nitrogen	89.1	2.25
Total casein	78.0	1.23
Total whey protein	11.1	1.70
$\kappa \text {-}$casein	9.45	1.48
$\beta \text {-}$casein	32.3	2.45
$\alpha _{S1}\text {-}$casein	25.7	1.79
$\alpha _{S2}\text {-}$casein	9.20	1.14
$\beta \text {-}$lactoglobulin	8.68	1.56
$\alpha \text {-}$lactalbumin	2.39	0.50

^a^% total milk N

### Statistical modeling

Three statistical approaches were used to combine multiple sources of information to predict nine phenotypes related to milk protein composition.

#### Multiple kernel learning

Kernel methods regress the phenotype on a kernel relationship matrix that is constructed using biological profiles of animals [23]. The model considered for the milk spectral data was:

1 $$\begin{aligned} {\mathbf {y}} = {\mathbf {Xb}} + {\mathbf {Z}}_{\text{IR}} {\mathbf {u}}_{\text{IR}} + {\mathbf {e}}, \end{aligned}$$

where ${\mathbf {y}}$ is the vector of phenotypic records, ${\mathbf {X}}$ is the design matrix for the on-farm data, ${\mathbf {b}}$ is the vector of the on-farm fixed effects, ${\mathbf {Z}}_{\text{IR}}$ is the incidence matrix relating animals to phenotypic records, $\mathbf {u_{\text{IR}}}$ is the vector of the random milk spectra values of the animals, and ${\mathbf {e}}$ is the vector of the residuals. The distributions of the random effects for the milk spectra and the residuals were assumed to follow ${\mathbf {u}}_{\text{IR}} \sim {\text{N}({\mathbf {0}},{\mathbf {S}} \sigma ^2_{\text{u}_{\text{IR}}})}$ and ${\mathbf {e}} \sim {\text{N}({\mathbf {0}},{\mathbf {I}} \sigma ^2_{\text{e}})}$, respectively, where $\sigma^2_{\text{u}_\text{IR}}$ is the spectral variance, $\sigma ^2_{\text{e}}$ is the residual variance, ${\mathbf {S}}$ is the spectra-based relationship matrix, and ${\mathbf {I}}$ is an identity matrix. The spectral-based relationship matrix, which represents the similarity of milk FTIR among individuals, was computed as a function of the spectral wavenumber cross-product:

2 $$\begin{aligned} {\mathbf {S}} = \frac{{\mathbf {W}}_{\text{IR}}\mathbf {W'}_{\text{IR}}}{{\textit{m}}_ {\text {IR}}}, \end{aligned}$$

where ${\mathbf {W}}_{\text{IR}}$ is the centered and standardized wavenumber matrix and ${m_\text {IR}} = 1060$ is the total number of wavenumbers. The construction of the ${\mathbf {S}}$ matrix followed that of the genomic relationship matrix of VanRaden [24]. The only difference was the source of information to create the relationship matrix.

Genetic markers were integrated by extending the above Eq. (1) via multiple kernel learning as follows.

3 $$\begin{aligned} {\mathbf {y}} = {\mathbf {Xb}} + {\mathbf {Z}}_{\text{IR}} {\mathbf {u}}_{\text{IR}} + {\mathbf {Z}}_{\text{g}} {\mathbf {u}}_{\text{g}} + {\mathbf {e}}, \end{aligned}$$

where ${\mathbf {Z}}_{\text{g}}$ is the incidence matrix for the additive genetic effects and $\mathbf {u_{\text{g}}}$ is the vector of the random additive genetic effects. The distribution of the random additive genetic effects was assumed to follow ${\mathbf {u}}_{\text{g}} \sim {\text{N}({\mathbf {0}},{\mathbf {G}} \sigma ^2_{\text{u}_{\text{g}}})}$, where $\sigma ^2_{{\text{u}}_{\text{g}}}$ is the variance of the additive genetic effects and ${\mathbf {G}}$ is the first genomic relationship matrix proposed by VanRaden [24]. Pedigree information was considered as an alternative source of genetic information. The genomic relationship matrix was then replaced with the numerator relationship matrix in Eq. (3) so that ${\mathbf {u}}_{\text{p}} \sim {\text{N}({\mathbf {0}},{\mathbf {A}} \sigma ^2_{\text{u}_{\text{p}}})}$. Here, ${\mathbf {A}}$ is the pedigree-based kinship relationship matrix and $\sigma ^2_{{\text{u}}_{\text{p}}}$ is the pedigree-based variance of the additive genetic effects.

#### Multilayer Bayesian variable selection

BayesB [25] was used to fit the model including only spectral information.

$$\begin{aligned} {\mathbf {y}} = {\mathbf {Xb}} + {\sum ^{{\text{m}}_\text{IR}}_{i=1}} {\mathbf {W}}_{{\text{IR}_\textit{i}}} \textit{a}_{{{\text{IR}}}_\textit{i}} + {\mathbf {e}}, \end{aligned}$$

where ${a_{{\text{IR}}_{i}}}$ is the *i*th wavenumber effect. A Gaussian prior with large variance was assigned to ${\mathbf {b}}$. The prior distribution of the *i*th wavenumber effect

$$\begin{aligned} p(\textit{a}_{{{\text{IR}}}_\textit{i}}| \pi ,\textit{df, S})= \pi \times t(\textit{a}_{{{\text{IR}}}_\textit{i}}|df, S)+(1-\pi ) \times (\textit{a}_{{{\text{IR}}}_\textit{i}}=0), \end{aligned}$$

where $\pi$ is the proportion of nonzero wavenumber effects and $t({a_{{\text{IR}}_{i}}}|df, S)$ is a scaled-t density with two hyperparameters, degrees of freedom, *df*, and scale, *S*. The residual variance was assigned a scaled-inverse $\chi ^2$ density with degrees of freedom, $df_e$, and scale, $S_e$ [5].

This BayesB model was further extended to multilayer BayesB by adding a separate mixture prior for the SNP term.

$$\begin{aligned} {\mathbf {y}} = \mathbf {Xb} + {{\sum ^{{\text{m}}_{\text {IR}}}}_{\textit{i=1}}} {\mathbf {W}}_{{{\text{IR}}}_\textit{i}} \textit{a}_{{\text{IR}}_\textit{i}} + {\sum ^{{\text{m}}_{\text {SNP}}}}_{\textit{j}=1} {\mathbf {W}}_{{\text{SNP}}_\textit{j}} \textit{a}_{{\text{SNP}}_\textit{j}} + {\mathbf {e}}, \end{aligned}$$

where $\mathbf {W}_{\text{SNP}_{j}}$ is a vector of the centered and scaled genotypes at the *j*th SNP and ${a}_{\text{SNP}_{j}}$ is the corresponding SNP effect. The prior distribution of the SNP effects followed that of the wavenumbers:

$$\begin{aligned} p(\textit{a}_{\text{SNP}_\textit{j}}|\pi ,\text{df}, \text{S})= \pi \times t(\textit{a}_{\text{SNP}_\textit{j}}|df, S)+(1-\pi ) \times (\textit{a}_{\text{SNP}_\textit{j}}=0). \end{aligned}$$

Multiple kernel learning and multilayer BayesB were implemented using the BGLR R package [26]. In multilayer BayesB, wavenumber hyperparameters were specified using the default rule in the package following Ferragina et al. [5]. For the SNP effects,“probin = 0.5” and “count = 10” were set to assign a Beta-prior for two fixed shape parameters and derived the proportion of nonzero SNP effects $\pi$ for the SNP term [26]. The two shape parameters of the beta distribution $\pi _0$ and $p_0$ were set to $\tfrac{0.5*(1-0.5)}{(10+1)}=0.023$ and 0.5, respectively. A total of 50,000 Markov Chain Monte Carlo samples after 50,000 burn-in with the thinning rate of 10 were used to obtain the posterior means for all unknowns.

#### Partial least squares

Partial least squares is one of the most common methods for spectral analysis and was used in this study as a baseline model. Unlike unsupervised principal component regression, PLS finds the latent variables that maximize the covariance between the predictors and the phenotypes while minimizing the error. This method was recently used to integrate spectral and genomic information [11, 19]. The mixOmics R package [27] was used to implement PLS. The optimum number of latent variables was determined using the root mean squared error with a maximum value of 50.

### Evaluation of model performance

Table 2 displays the list of prediction models examined in this study. Model 1 (M1) was considered the baseline and included only spectral data. Both on-farm and genetic data were sequentially added to evaluate the gain from M1. Model 2 (M2) included herd and spectral effects because recent studies showed that herd information may greatly impact prediction performance. Model 3 (M3) included herd, DIM, parity, and spectral effects. Model 4 (M4) included herd, DIM, parity, milk spectra, and SNP data. M1 to M4 were fit across multiple kernel learning, multilayer BayesB, and PLS. Model 5 (M5) and Model 6 (M6) were applied to multiple kernel learning to make it more akin to multilayer BayesB by performing variable selection via association analysis. M5 included herd, DIM, parity, milk spectra, and the top three markers identified by genome-wide association analysis. The inclusion of markers with a large effect as fixed effects might improve prediction accuracy [28] because kernel methods weigh SNPs equally in the construction of a genomic relationship matrix. M4 was used to select the top three SNPs from the training data in cross-validation, as described later. M6 is the same as M5 except for the presence of a genomic relationship matrix constructed using all the markers excluding the top three SNPs. The top three SNPs were not considered in multilayer BayesB because this method performs variable selection internally. Pedigree information was used in Model 7 (M7) along with herd, DIM, parity, and spectral data assuming a practical situation where cow genotype data is not available for all individuals on a dairy farm. M7 was fit using multiple kernel learning and PLS.

**Table 2:** A list of covariates included in multiple kernel learning, multilayer BayesB, and partial least squares (PLS)

Model	Sub-model	Effect^a^						
		Herd	DIM	Parity	FTIR	Genomics	Top markers	Pedigree
Kernel	M1				$\checkmark$			
	M2	$\checkmark$			$\checkmark$			
	M3	$\checkmark$	$\checkmark$	$\checkmark$	$\checkmark$			
	M4	$\checkmark$	$\checkmark$	$\checkmark$	$\checkmark$	$\checkmark$		
	M5	$\checkmark$	$\checkmark$	$\checkmark$	$\checkmark$		$\checkmark$	
	M6	$\checkmark$	$\checkmark$	$\checkmark$	$\checkmark$	$\checkmark$	$\checkmark$	
	M7	$\checkmark$	$\checkmark$	$\checkmark$	$\checkmark$			$\checkmark$
BayesB	M1				$\checkmark$			
	M2	$\checkmark$			$\checkmark$			
	M3	$\checkmark$	$\checkmark$	$\checkmark$	$\checkmark$			
	M4	$\checkmark$	$\checkmark$	$\checkmark$	$\checkmark$	$\checkmark$		
PLS	M1				$\checkmark$			
	M2	$\checkmark$			$\checkmark$			
	M3	$\checkmark$	$\checkmark$	$\checkmark$	$\checkmark$			
	M4	$\checkmark$	$\checkmark$	$\checkmark$	$\checkmark$	$\checkmark$		
	M7	$\checkmark$	$\checkmark$	$\checkmark$	$\checkmark$			$\checkmark$

^a^DIM: days in milk; FTIR: milk Fourier transform infrared spectroscopy; Top markers: top three markers with the largest effects; Genomics: genomic relationship matrix in kernel methods, markers in BayesB, and principal components of genomic relationship matrix in PLS; Pedigree: numerator relationship matrix in kernel methods and principal components of numerator relationship matrix in PLS

### Cross-validation

Two CV scenarios repeated ten times were employed to assess model predictive performance for nine milk protein component traits. The phenotypes in the testing set were predicted by fitting multiple kernel learning, multilayer BayesB, and PLS in order to investigate the relative contributions of different sources of information according to Table 2. Predictive performance was evaluated using the prediction coefficient of determination (${\text{R}}^2$), which was calculated as the square of the correlation between the observed and predicted values in the testing set. The regression coefficient was also calculated by regressing the observed phenotypes of the individuals in the testing set on predicted values.

#### Repeated random sub-sampling cross-validation

We partitioned the data into training and testing sets of 716 and 250 cows, respectively. This CV was used because of a relatively small sample size. The predictive values of the testing set individuals ${\hat{\mathbf {y}}}_{\text{tst}}$ were predicted using estimated spectra, on-farm data, or/and genetic effects in the following manner. The conditional expectation of $E({\hat{\mathbf {y}}}_{\text{tst}} | {\hat{\mathbf {y}}}_{\text{trn}})$ was computed in kernel methods as ${\mathbf {S}}_{\text{tst},\text{trn}} {\mathbf {S^{-1}}}_{\text{trn},{\text{trn}}} {\hat{\mathbf {u}}}_{\text{IR}_{\text{trn}}}$ in M1, ${\mathbf {X}}_{\text{tst}} {\hat{\mathbf {b}}}_{{\text{trn}}} + {\mathbf {S}}_{\text{tst},\text{trn}} {\mathbf {S^{-1}}}_{\text{trn},{\text{trn}}} {\hat{\mathbf {u}}}_{\text{IR}_{\text{trn}}}$ in M2 and M3, ${\mathbf {X}}_{\text{tst}} {\hat{\mathbf {b}}}_{{\text{trn}}} + {\mathbf {S}}_{\text{tst},{\text{trn}}} {\mathbf {S^{-1}}}_{\text{trn},\text{trn}} {\hat{\mathbf {u}}}_{\text{IR}_{\text{trn}}}+{\mathbf {G}}_{\text{tst},{\text{trn}}} {\mathbf {G^{-1}}}_{\text{trn},\text{trn}} {\hat{\mathbf {u}}}_{\text{g}_{\text{trn}}}$ in M4, ${\mathbf {X}}_{\text{tst}} {\hat{\mathbf {b}}}_{{\text{trn}}} + {\mathbf {S}}_{\text{tst},\text{trn}} {\mathbf {S^{-1}}}_{\text{trn},\text{trn}} {\hat{\mathbf {u}}}_{\text{IR}_{\text{trn}}}+ {\mathbf {W}}_{{\text{SNP}}3} {\hat{\mathbf {b}}}_{{\text{SNP}}3}$ in M5, ${\mathbf {X}}_{\text{tst}} {\hat{\mathbf {b}}}_{{\text{trn}}} + {\mathbf {S}}_{\text{tst},\text{trn}} {\mathbf {S^{-1}}}_{\text{trn},\text{trn}} {\hat{\mathbf {u}}}_{\text{IR}_{\text{trn}}}+{\mathbf {G}}_{\text{tst},\text{trn}} {\mathbf {G^{-1}}}_{\text{trn},{\text{trn}}} {\hat{\mathbf {u}}}_{\text{g}_{\text{trn}}} + {\mathbf {W}}_{\text{SNP}3} {\hat{\mathbf {b}}}_{\text{SNP}3}$ in M6, and ${\mathbf {X}}_{\text{tst}} {\hat{\mathbf {b}}}_{{\text{trn}}} + {\mathbf {S}}_{\text {tst},{\text{trn}}} {\mathbf {S^{-1}}}_{\text{trn},\text{trn}} {\hat{\mathbf {u}}}_{\text{IR}_{\text{trn}}}+{\mathbf {A}}_{\text{tst},\text{trn}} {\mathbf {A^{-1}}}_{\text{trn},\text{trn}} {\hat{\mathbf {u}}}_{\text{p}_{\text{trn}}}$ in M7. Here, ${\mathbf {X}}_{\text{tst}}$ and ${\hat{\mathbf {b}}}_{{\text{trn}}}$ are the design matrix for on-farm data in the testing individuals and the corresponding effects estimated from the training set, respectively. And, $\mathbf {S}_{\text{tst},\text{trn}}$, $\mathbf {G}_{\text{tst},\text{trn}}$ and $\mathbf {A}_{\text{tst},\text{trn}}$ are relationship matrices between the testing and training individuals according to their spectra, genomic, and pedigree profiles, respectively. Similarly, $\mathbf {S^{-1}}_{\text{trn},\text{trn}}$, $\mathbf {G^{-1}}_{\text{trn},\text{trn}}$ and $\mathbf {A^{-1}}_{\text{trn},\text{trn}}$ are the inverse of relationship matrices between the individuals in the training set according to their spectra, genomic, and pedigree profiles, respectively. The vectors of ${\hat{\mathbf {u}}}_{\text{IR}_{\text{trn}}}$, ${\hat{\mathbf {u}}}_{\text{g}_{\text{trn}}}$, and ${\hat{\mathbf {u}}}_{\text{p}_{\text{trn}}}$ are predicted spectral, additive genomic, and additive genetic values, respectively. The marker matrix ${\mathbf {W}}_{\text{SNP}3}$ includes the top three markers based on the absolute values of their marker effects and ${\hat{\mathbf {b}}}_{\text{SNP}3}$ is the vector of corresponding marker effects. In BayesB, ${\hat{\mathbf {y}}}_\text{tst}$ was obtained as ${\mathbf {W}}{_{\text{IR}_{\text{tst}}}} {\hat{\mathbf {a}}}_{\text{IR}_{\text {trn}}}$ in M1, $\mathbf {X}_{\text{tst}} {\hat{\mathbf {b}}}_{{\text{trn}}} + \mathbf {W}{_{\text{IR}_{\text{tst}}}} {\hat{\mathbf {a}}}_{\text{IR}_{\text {trn}}}$ in M2 and M3, $\mathbf {X}_{\text{tst}} {\hat{\mathbf {b}}}_{{\text{trn}}} + \mathbf {W}{_{\text{IR}_{\text{tst}}}} {\hat{\mathbf {a}}}_{\text{IR}_{\text {trn}}} + \mathbf {W}{_{\text{SNP}_{\text{tst}}}} {\hat{\mathbf {a}}}_{\text{SNP}_{\text {trn}}}$ in M4. Here, $\mathbf {W}{_{\text{IR}_{\text{tst}}}}$ and $\mathbf {W}{_{\text{snp}_{\text{tst}}}}$ are the incidence matrices of the testing set individuals for wavenumbers and markers, respectively, and ${\hat{\mathbf {a}}}_{\text{IR}_{\text {trn}}}$ and ${\hat{\mathbf {a}}}_{\text{snp}_{\text {trn}}}$ are the vectors of wavenumber and marker effects, respectively, obtained from the training set. In PLS, the principal components of ${\mathbf {G}}$ and ${\mathbf {A}}$ were extracted when fitting M4 and M7. The first 115 and 152 principal components of ${\mathbf {G}}$ and ${\mathbf {A}}$ were used, which explained over 80% of the variation. In PLS, the prediction was performed as ${\mathbf {Q}}_{\text {tst}} {\hat{\mathbf {q}}}_{\text {trn}}$, where ${\mathbf {Q}}_{\text {tst}}$ is the principal component matrix extracted from the spectra, on-farm, or/and genetic covariates of the individuals in the testing set and ${\hat{\mathbf {q}}}_{\text {trn}}$ is the vector of corresponding principal component effects estimated from the training set. The optimal number of principal components was determined in the training set and the same number of principal components was extracted in the testing set.

#### Herd cross-validation

 The repeated random sub-sampling CV may result in over-prediction due to the dependency between herd and spectra variability [5, 7]. For this reason, herd CV, which is a random-sampling method based on herds, was explored to exclude the possibility of over-prediction. We randomly assigned 65 and 20 herds as training and testing sets, respectively. The number of individuals in the testing set in each run ranged from 223 to 251. In herd CV, the herd effect was not included in the aforementioned seven models. Thus, M2 with herd and spectral effects was not considered.

## Results

### Repeated random sub-sampling cross-validation

**Fig. 1:**
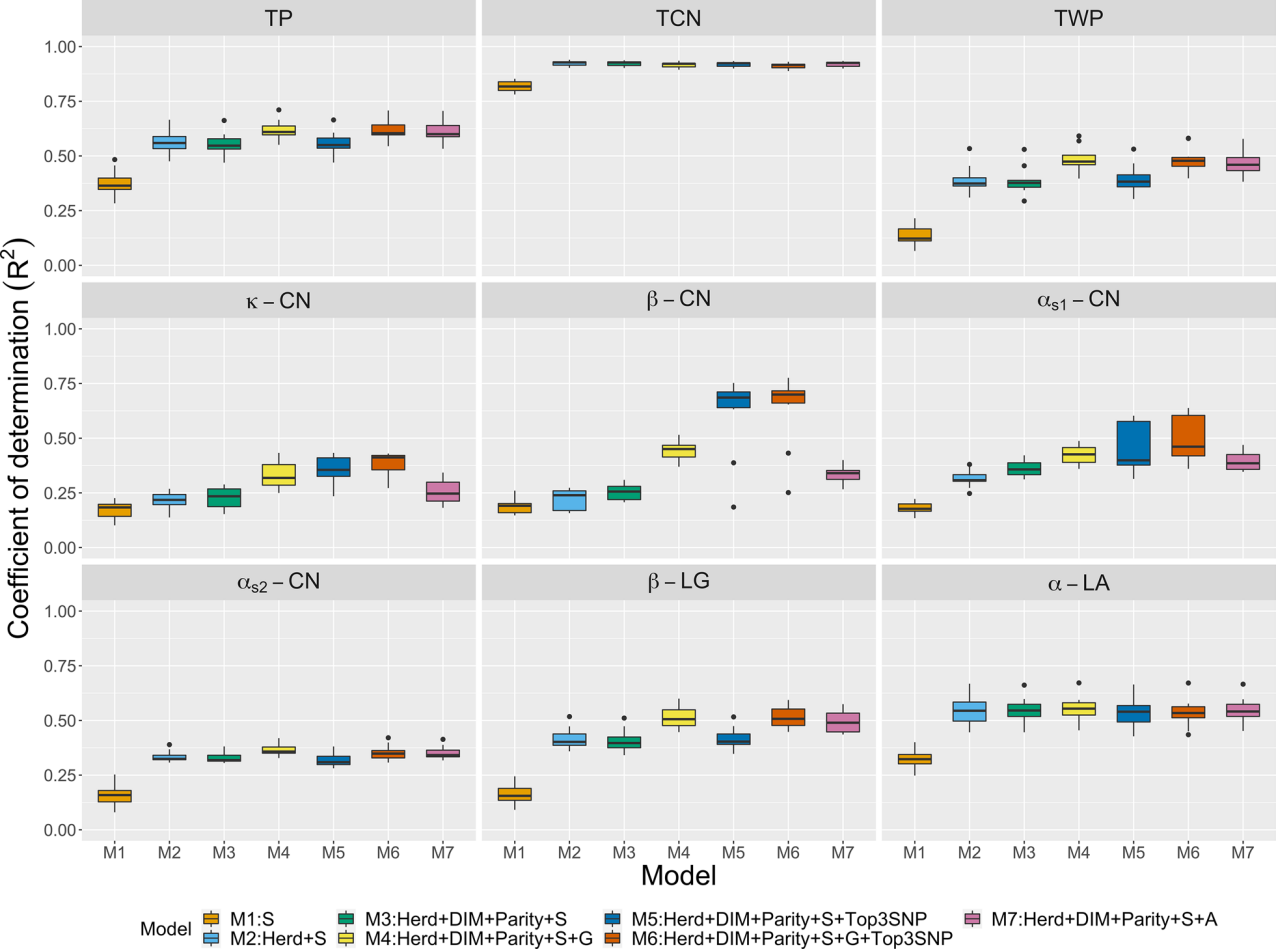
Prediction R-squared for milk protein traits (TP: true protein nitrogen; TCN: total casein; TWP: total whey protein; $\kappa \text {-}$CN: $\kappa \text {-}$casein; $\beta \text {-}$CN: $\beta \text {-}$casein; $\alpha _{S1}\text {-}$CN: $\alpha _{S1}\text {-}$casein; $\alpha _{S2}\text {-}$CN: $\alpha _{S2}\text {-}$casein; $\beta \text {-}$LG: $\beta \text {-}$lactoglobulin; $\alpha \text {-}$LA: $\alpha \text {-}$lactalbumin) from multiple kernel learning using repeated random sub-sampling cross-validation. ${\mathbf {S}}$: spectral relationship matrix; ${\mathbf {G}}$: genomic relationship matrix; ${\mathbf {A}}$: numerator relationship matrix; DIM: days in milk; Top3SNP: top three markers with the largest effects.

Figure 1 shows $\text{R}^2$ values obtained by multiple kernel learning using repeated random sub-sampling CV. The model including spectra only (M1) produced the lowest $\text{R}^2$ ranging from 0.14 to 0.82. When on-farm predictors were added to the models (herd in M2; herd, DIM, and parity in M3), $\text{R}^2$ increased, except for $\beta \text {-}$CN, ranging from 0.20 to 0.92. The small difference observed in $\text{R}^2$ between M2 and M3 indicates that DIM and parity made only small contributions compared to the herd effect. Joint modeling of spectra, on-farm information, and genomic data (M4) further improved $\text{R}^2$ compared with M1 to M3 ranging from 0.31 to 0.91. In particular, prediction of $\beta \text {-}$CN was markedly improved. $\text{R}^2$ was increased by fitting the top three markers as fixed effects (M5 and M6) for $\kappa \text {-}$CN and $\beta \text {-}$CN. However, prediction performance for the other traits did not clearly improve. Furthermore, $\text{R}^2$ values from pedigree (M7) were higher than those for M1 to M3 but lower than or similar to that for M4. Therefore, considering genomic or pedigree information may improve the ability of a model to predict most milk protein traits. The regression coefficients obtained by using multiple kernel learning are listed in Table 3. M1 slightly underestimated predictions, with slope values ranging from 0.99 to 1.12. In contrast, M2 to M7 showed a tendency for over-prediction, with slope values ranging from 0.74 to 1.01. When genomic or pedigree data were added (M4 to M7), the extent of bias was smaller compared to those of M2 and M3.

**Table 3:** Regression coefficients of predictive values for testing cows on observed phenotypes when fitting multiple kernel learning, multilayer BayesB, and partial least squares (PLS) using repeated random sub-sampling cross-validation

Traits	Kernel							BayesB				PLS				
	M1	M2	M3	M4	M5	M6	M7	M1	M2	M3	M4	M1	M2	M3	M4	M7
True protein nitrogen	1.04	0.92	0.91	0.94	0.90	0.93	0.94	0.96	0.92	0.91	0.93	0.87	0.89	0.87	0.92	0.88
Total casein	1.02	1.01	1.01	1.01	1.01	1.00	1.01	0.98	0.97	0.96	0.99	0.98	0.98	0.97	0.97	0.98
Total whey protein	1.01	0.83	0.81	0.87	0.81	0.86	0.86	0.96	0.85	0.82	0.90	0.82	0.77	0.74	0.72	0.75
$\kappa \text {-}$casein	1.02	0.76	0.74	0.84	0.85	0.85	0.79	0.92	0.84	0.82	0.92	0.76	0.71	0.71	0.75	0.73
$\beta \text {-}$casein	1.12	0.78	0.78	0.92	0.93	0.94	0.86	0.97	0.84	0.82	0.96	0.83	0.83	0.86	0.85	0.84
$\alpha _{S1}\text {-}$casein	1.05	0.86	0.86	0.88	0.87	0.88	0.86	1.02	0.87	0.86	0.92	0.84	0.86	0.90	0.82	0.80
$\alpha _{S2}\text {-}$casein	1.02	0.84	0.83	0.83	0.80	0.79	0.83	0.99	0.84	0.83	0.85	0.79	0.78	0.78	0.66	0.64
$\beta \text {-}$lactoglobulin	0.99	0.84	0.83	0.88	0.83	0.88	0.87	0.99	0.85	0.85	0.90	0.76	0.75	0.78	0.77	0.73
$\alpha \text {-}$lactalbumin	1.06	0.92	0.92	0.92	0.89	0.88	0.92	1.02	0.92	0.92	0.92	0.94	0.91	0.91	0.93	0.91

M1: milk Fourier transform infrared spectroscopy (FTIR)

M2: herd + FTIR

M3: herd + days in milk + parity + FTIR

M4: herd + days in milk + parity + FTIR + Genomics

M5: herd + days in milk + parity + FTIR + top three markers with the largest effects

M6: herd + days in milk + parity + FTIR + Genomics + top three markers with the largest effects

M7: herd + days in milk + parity + FTIR + pedigree

**Fig. 2:**
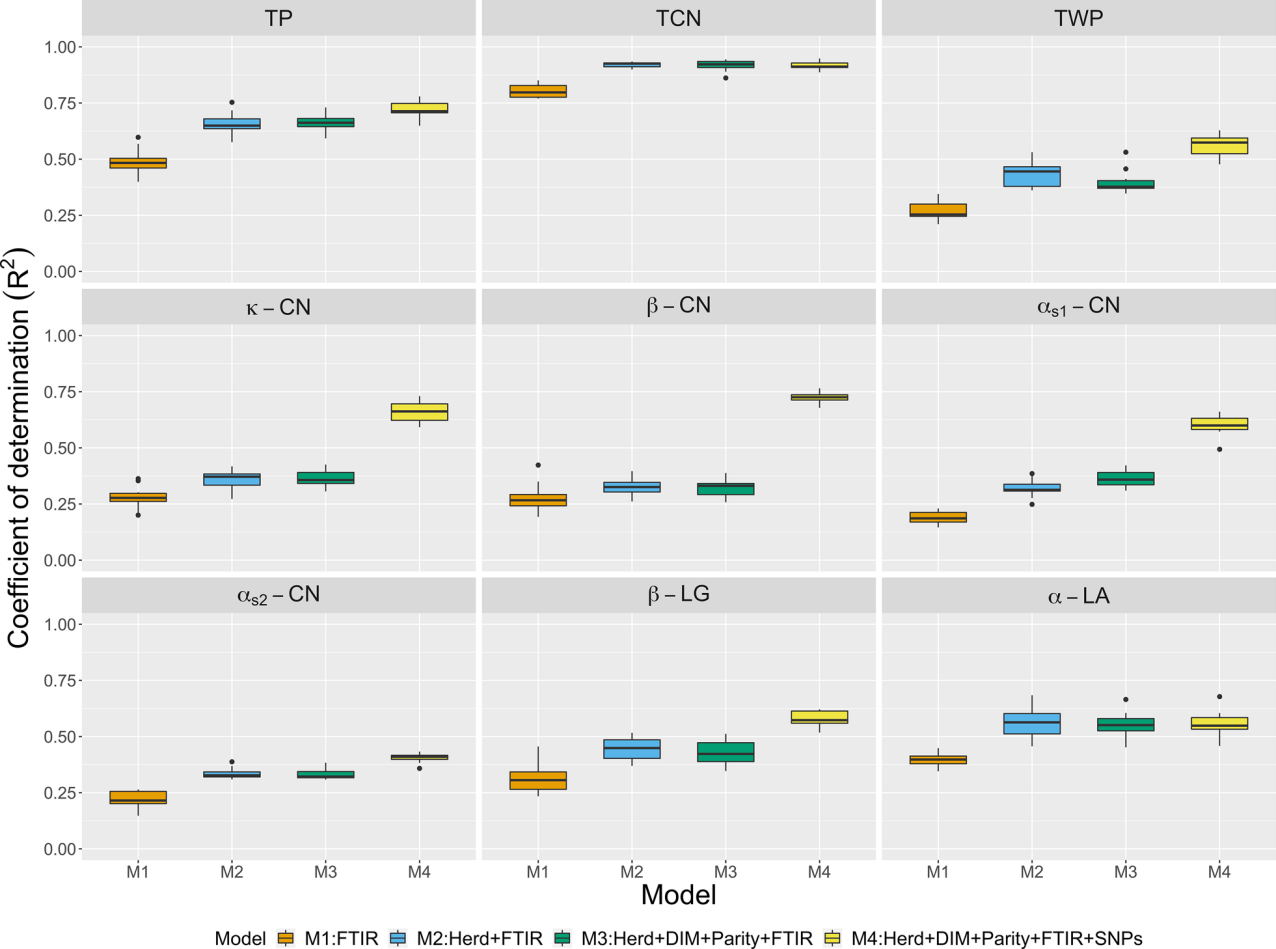
Prediction R-squared for milk protein traits (TP: true protein nitrogen; TCN: total casein; TWP: total whey protein; $\kappa \text {-}$CN: $\kappa \text {-}$casein; $\beta \text {-}$CN: $\beta \text {-}$casein; $\alpha _{S1}\text {-}$CN: $\alpha _{S1}\text {-}$casein; $\alpha _{S2}\text {-}$CN: $\alpha _{S2}\text {-}$casein; $\beta \text {-}$LG: $\beta \text {-}$lactoglobulin; $\alpha \text {-}$LA: $\alpha \text {-}$lactalbumin) from multilayer BayesB using repeated random sub-sampling cross-validation. FTIR: milk Fourier transform infrared spectroscopy; DIM: days in milk.

Figure 2 shows $\text{R}^2$ values from multilayer BayesB. The model with spectra only (M1) yielded the lowest prediction for all traits. Note that $\text{R}^2$ was improved when on-farm data were added to M1 (M2 to M3). Inclusion of the SNP data (M4) via multilayer BayesB produced the highest $\text{R}^2$. Large improvements in $\text{R}^2$ were observed for $\kappa \text {-}$CN , $\beta \text {-}$CN, and $\alpha _{S1}\text {-}$CN. For all traits, multilayer BayesB yielded higher $\text{R}^2$ than multiple kernel learning. All regression coefficients were less than 1 except for $\alpha _{S1}\text {-}$CN and $\alpha \text {-}$LA in M1 (Table 3). Moreover, bias was reduced by including genomic information.

**Fig. 3:**
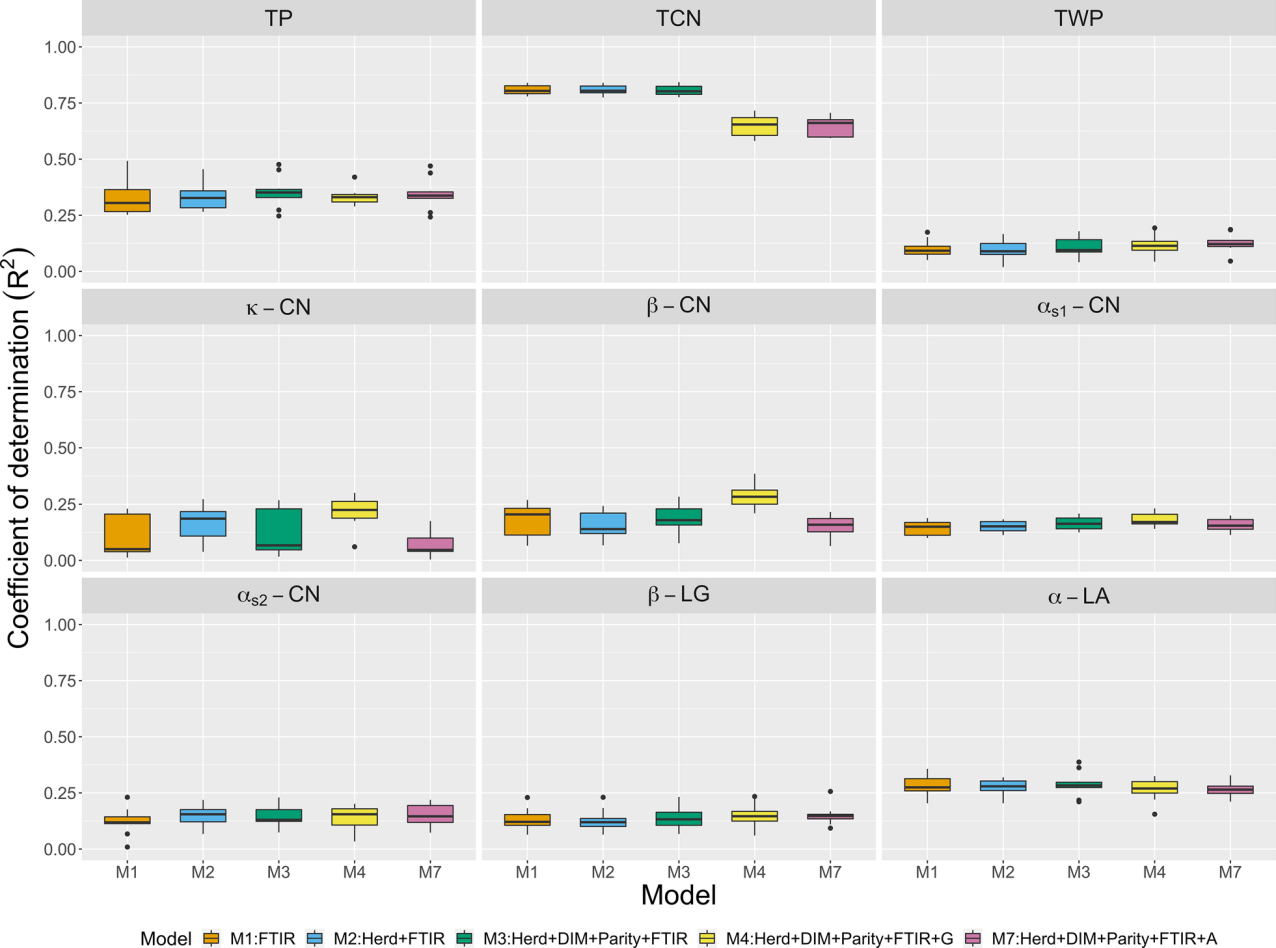
Prediction R-squared for milk protein traits (TP: true protein nitrogen; TCN: total casein; TWP: total whey protein; $\kappa \text {-}$CN: $\kappa \text {-}$casein; $\beta \text {-}$CN: $\beta \text {-}$casein; $\alpha _{S1}\text {-}$CN: $\alpha _{S1}\text {-}$casein; $\alpha _{S2}\text {-}$CN: $\alpha _{S2}\text {-}$casein; $\beta \text {-}$LG: $\beta \text {-}$lactoglobulin; $\alpha \text {-}$LA: $\alpha \text {-}$lactalbumin) from partial least squares using repeated random sub-sampling cross-validation. FTIR: milk Fourier transform infrared spectroscopy; ${\mathbf {G}}$: Principal components of genomic relationship matrix; ${\mathbf {A}}$: Principal components of numerator relationship matrix; DIM: days in milk.

Figure 3 presents $\text{R}^2$ values for the five alternative covariate sets (M1 to M4 and M7) fitted with PLS. In this case, $\text{R}^2$ did not improve by adding on-farm, genomic, or pedigree information to spectra. In fact, genetic information even lowered $\text{R}^2$ for certain traits. All regression coefficients were less than 1 (Table 3). The difference in prediction performance among multiple kernel learning, multilayer BayesB, and PLS was small for M1. However, multiple kernel learning and multilayer BayesB outperformed PLS when either on-farm data or genetic data were considered (M2 to M7). Note that, in most cases, multilayer BayesB performed better than multiple kernel learning.

### Herd cross-validation

**Fig. 4:**
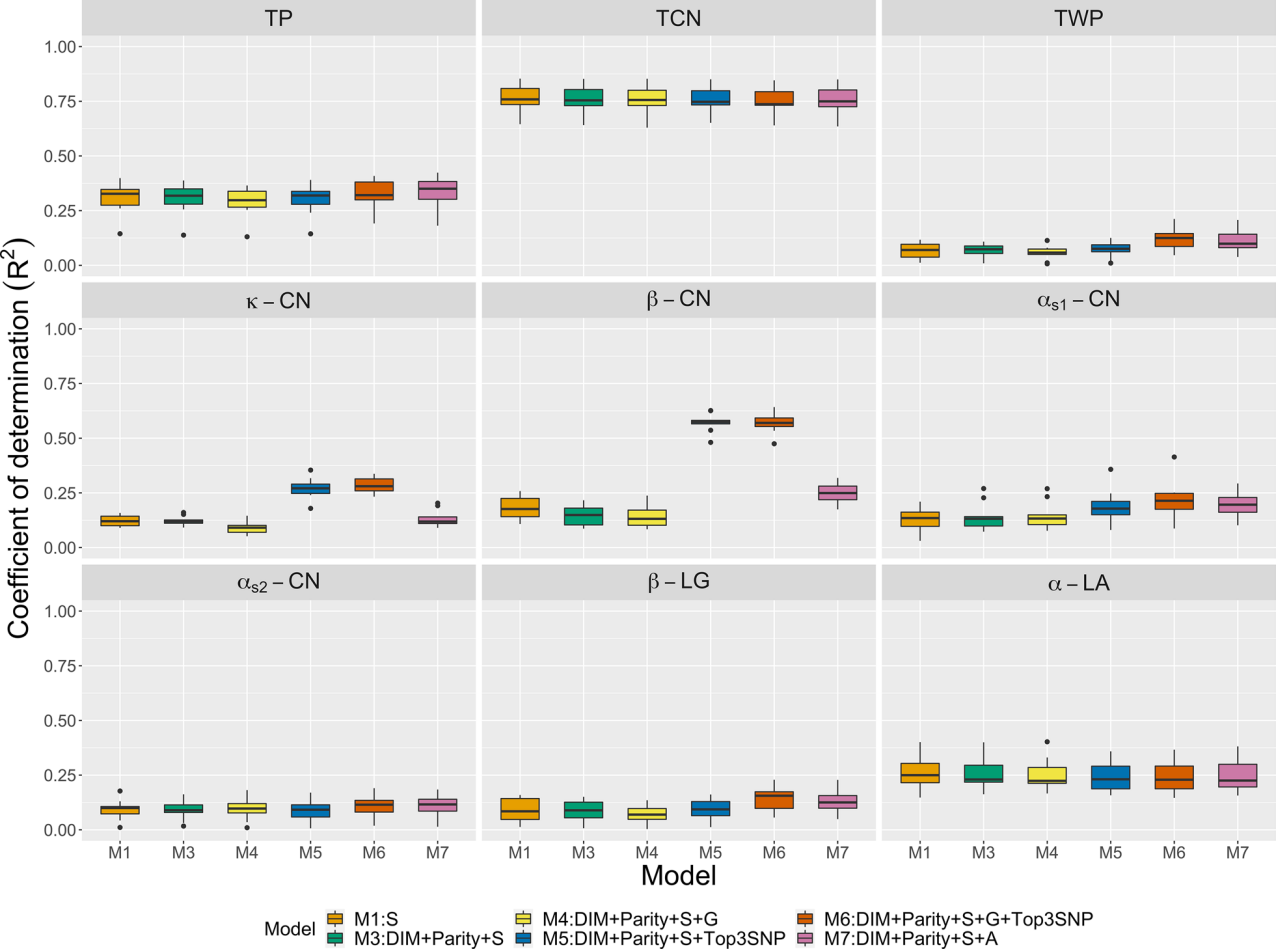
Prediction R-squared for milk protein traits (TP: true protein nitrogen; TCN: total casein; TWP: total whey protein; $\kappa \text {-}$CN: $\kappa \text {-}$casein; $\beta \text {-}$CN: $\beta \text {-}$casein; $\alpha _{S1}\text {-}$CN: $\alpha _{S1}\text {-}$casein; $\alpha _{S2}\text {-}$CN: $\alpha _{S2}\text {-}$casein; $\beta \text {-}$LG: $\beta \text {-}$lactoglobulin; $\alpha \text {-}$LA: $\alpha \text {-}$lactalbumin) from multiple kernel learning using herd cross-validation. ${\mathbf {S}}$: spectral relationship matrix; ${\mathbf {G}}$: genomic relationship matrix; ${\mathbf {A}}$: numerator relationship matrix; DIM: days in milk; Top3SNP: top three markers with the largest effects.

**Fig. 5:**
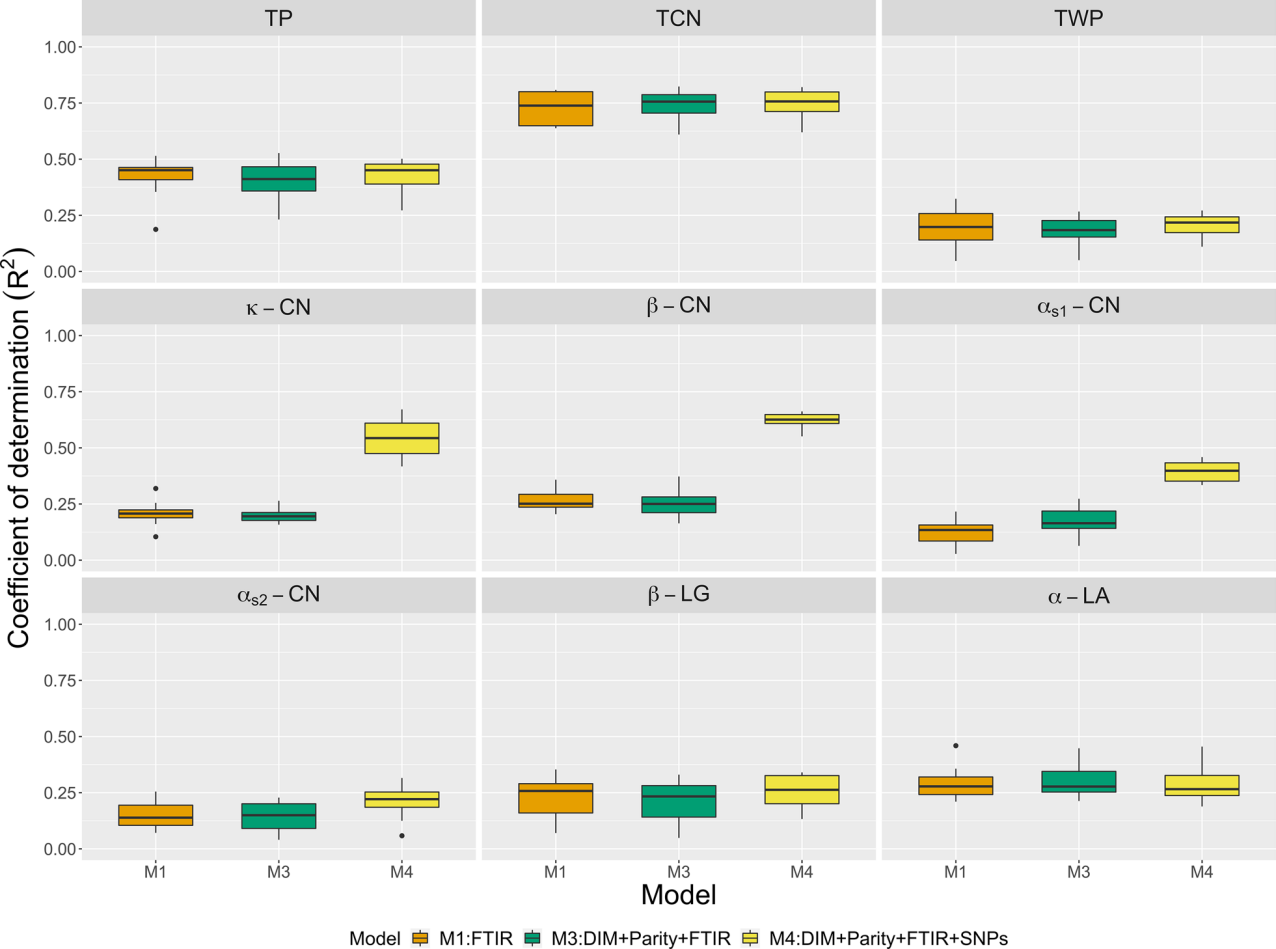
Prediction R-squared for milk protein traits (TP: true protein nitrogen; TCN: total casein; TWP: total whey protein; $\kappa \text {-}$CN: $\kappa \text {-}$casein; $\beta \text {-}$CN: $\beta \text {-}$casein; $\alpha _{S1}\text {-}$CN: $\alpha _{S1}\text {-}$casein; $\alpha _{S2}\text {-}$CN: $\alpha _{S2}\text {-}$casein; $\beta \text {-}$LG: $\beta \text {-}$lactoglobulin; $\alpha \text {-}$LA: $\alpha \text {-}$lactalbumin) from multilayer BayesB using herd cross-validation. FTIR: milk Fourier transform infrared spectroscopy; DIM: days in milk.

**Fig. 6:**
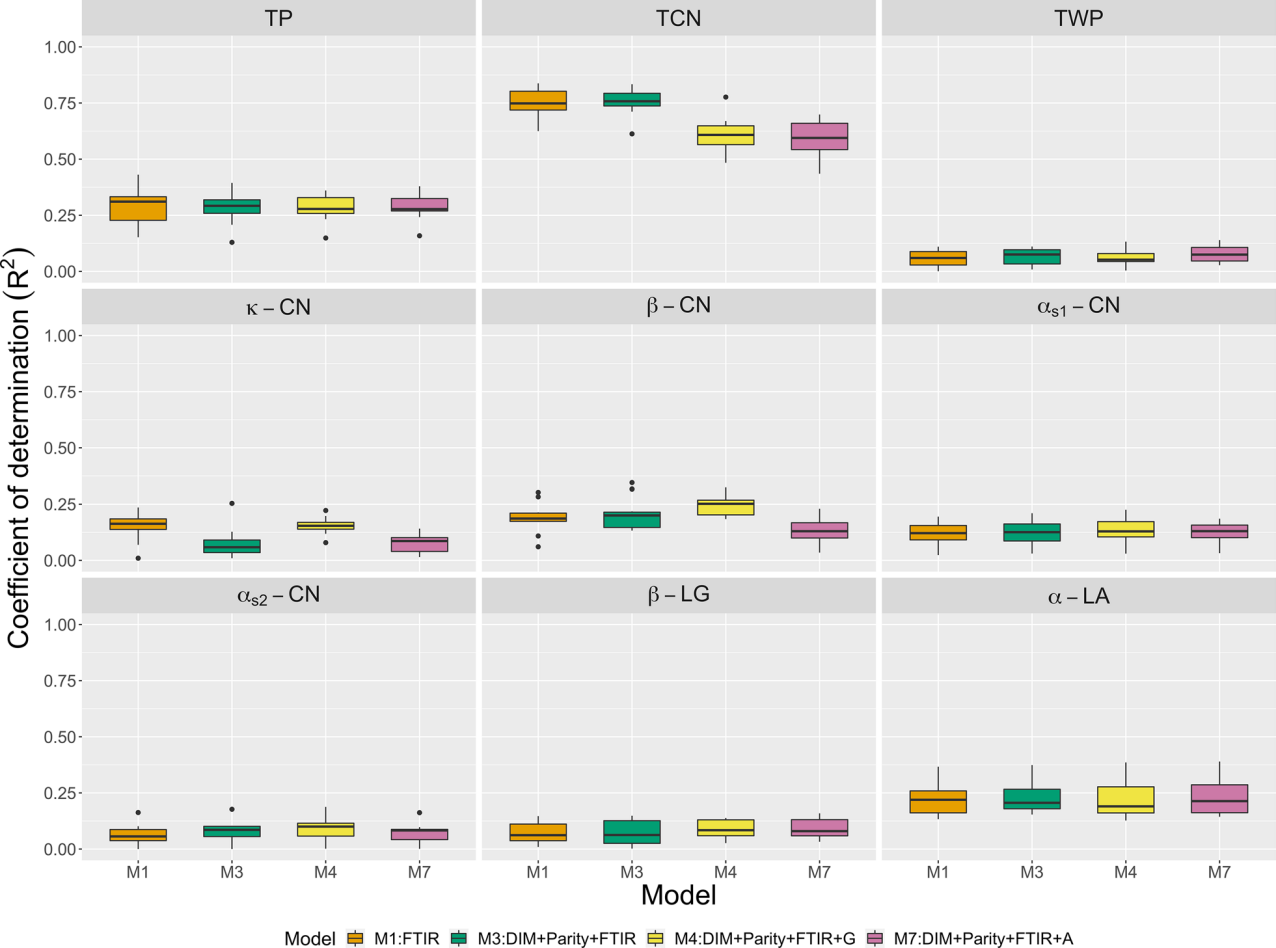
Prediction R-squared for milk protein traits (TP: true protein nitrogen; TCN: total casein; TWP: total whey protein; $\kappa \text {-}$CN: $\kappa \text {-}$casein; $\beta \text {-}$CN: $\beta \text {-}$casein; $\alpha _{S1}\text {-}$CN: $\alpha _{S1}\text {-}$casein; $\alpha _{S2}\text {-}$CN: $\alpha _{S2}\text {-}$casein; $\beta \text {-}$LG: $\beta \text {-}$lactoglobulin; $\alpha \text {-}$LA: $\alpha \text {-}$lactalbumin) from partial least squares using herd cross-validation. FTIR: milk Fourier transform infrared spectroscopy; ${\mathbf {G}}$: Principal components of genomic relationship matrix; ${\mathbf {A}}$: Principal components of numerator relationship matrix; DIM: days in milk.

Herd CV was designed to avoid the over-prediction caused by the known relationship between herd and spectral variability. We considered the same covariate sets as those used in the repeated random sub-sampling CV except for the herd effect. Figures 4, 5, and 6 present $\text{R}^2$ values produced by multiple kernel learning, multilayer BayesB, and PLS, respectively. As expected, herd CV showed lower $\text{R}^2$ than the repeated random sub-sampling CV. In multiple kernel learning, the model including on-farm DIM and parity data (M3) did not provide better predictions than the model including spectra only (M1) (Fig. 4). Jointly fitting genomic and spectral relationship matrices (M4) did not increase $\text{R}^2$. Nevertheless, $\text{R}^2$ markedly increased for $\kappa \text {-}$CN and $\beta \text {-}$CN when the top three markers were included in the models (M5 and M6). In contrast, the addition of SNP data to the spectral data improved $\text{R}^2$ for multilayer BayesB (M4) (Fig. 5). The genomic data did not increase $\text{R}^2$ for PLS (Fig. 6). In multiple kernel learning, the pedigree data slightly improved $\text{R}^2$ compared to that obtained using the spectra only. However, no increase in $\text{R}^2$ was observed for PLS. The herd CV regression coefficients are presented in Table 4. There was upward bias in all models but the kernel methods and BayesB showed less bias than PLS. Whereas adding on-farm and genomic information (M3 and M4) in multiple kernel learning showed greater bias than M1 for many of the fraction traits, the extent of bias was smaller in multilayer BayesB.

**Table 4:** Regression coefficients of predictive value for testing cows on phenotype when fitting multiple kernel learning, multilayer BayesB, and partial least squares (PLS) using herd cross-validation

Traits	Kernel						BayesB			PLS			
	M1	M3	M4	M5	M6	M7	M1	M3	M4	M1	M3	M4	M7
True protein nitrogen	0.90	0.89	0.88	0.88	0.87	0.90	0.84	0.86	0.87	0.30	0.31	0.28	0.29
Total casein	0.96	0.96	0.96	0.96	0.95	0.96	0.89	0.92	0.91	0.75	0.74	0.62	0.58
Total whey protein	0.71	0.69	0.64	0.68	0.74	0.74	0.73	0.77	0.74	0.06	0.05	0.08	0.07
$\kappa \text {-}$casein	0.83	0.79	0.84	0.94	0.94	0.77	0.79	0.80	0.93	0.14	0.14	0.15	0.05
$\beta \text {-}$casein	0.96	0.77	0.82	0.97	0.96	0.92	0.86	0.88	0.95	0.21	0.18	0.26	0.13
$\alpha _{S1}\text {-}$casein	0.84	0.68	0.71	0.77	0.83	0.85	0.79	0.85	0.90	0.12	0.10	0.13	0.12
$\alpha _{S2}\text {-}$casein	0.78	0.70	0.74	0.68	0.73	0.77	0.77	0.75	0.84	0.08	0.09	0.10	0.07
$\beta \text {-}$lactoglobulin	0.75	0.71	0.64	0.68	0.74	0.75	0.78	0.78	0.81	0.07	0.07	0.08	0.08
$\alpha \text {-}$lactalbumin	0.91	0.92	0.94	0.84	0.82	0.88	0.80	0.87	0.84	0.22	0.22	0.22	0.22

M1: milk Fourier transform infrared spectroscopy (FTIR)

M3: days in milk (DIM)+ parity + FTIR

M4: DIM + parity + FTIR + genomics

M5: DIM + parity + FTIR + top three markers with the largest effects

M6: herd + DIM + parity + FTIR + genomics + top three markers with the largest effects

M7: herd + DIM + parity + FTIR + pedigree

## Discussion

### Predictive ability across dairy farms

Repeated random sub-sampling or k-fold CV has often been used to evaluate a calibration equation in spectral analysis. However, the $\text{R}^2$ derived from this type of CV may be over-optimistic as the records from the same herds could be included in both training and testing data [7, 11]. For instance, Wang and Bovenhuis [7] found that the $\text{R}^2$ for $\text{CH}_4$ emission was small when it was obtained from milk FTIR spectra using a CV scheme based on herds. In contrast, a moderate $\text{R}^2$ was observed for k-fold CV. In addition, Luke et al. [8] showed that prediction for serum metabolic concentrations was lower with herd CV than with k-fold CV. In our study, the $\text{R}^2$ from herd CV was lower than that obtained using repeated random sub-sampling CV. In routine evaluations, the use of herd CV must be carefully considered before developing a calibration model because repeated random subsampling or k-fold CV may provide over-optimistic predictions. Our herd CV results suggest that the use of genomic and pedigree information can be beneficial whereas on-farm information adds relatively less value.

### Integration of heterogeneous data

There is growing interest in exploiting multiple sources of information to perform spectral-based predictions of novel phenotypes [11, 19]. We analyzed the impact of using on-farm (herd, DIM, and parity), genomic, and pedigree information on forecasting milk protein composition. To this end, we applied multiple kernel learning and multilayer BayesB. Some previous studies investigated the predictive performance for milk protein composition expressed as g/L or g/dL of milk based on k-fold cross-validation using spectra alone. For example, Bonfatti et al. [13] and Rutten et al. [16] obtained low to moderate $\text{R}^2$ values that ranged from 0.31 to 0.78 and from 0.18 to 0.56, respectively. Bonfatti et al. [13, 29] reported that the $\text{R}^2$ values for milk protein composition expressed as g/L were higher than those for percentage protein. Our prediction of unit of total N content in the casein fractions using milk FTIR spectra showed lower $\text{R}^2$, ranging from 0.11 to 0.50 for repeated random sub-sampling CV and from 0.06 to 0.43 for herd CV. However, we found higher $\text{R}^2$ values for total casein (0.79 to 0.82 in repeated random sub-sampling CV; 0.73 to 0.76 in herd CV) than for the other traits evaluated. Hence, milk FTIR spectral information might suffice to predict total casein.

On-farm information plus milk FTIR spectra produced higher $\text{R}^2$ than that of the model with milk FTIR spectra alone in repeated random sub-sampling CV. In contrast, the results for herd CV showed only minor improvements relative to the milk FTIR spectra model. Therefore, the inclusion of these explanatory predictors may only be effective when they are applied to the same herds. We found no prior literature integrating milk FTIR spectra with on-farm data to predict milk protein components. Recent studies assessed the contribution of on-farm information to predict reproductive traits [10, 11]. In repeated random sub-sampling CV, adding the herd effect enhanced the prediction more effectively than adding DIM or parity. This finding was consistent with that of a previous study [10]. Indeed, Toledo-Alvarado et al. [10] reported that joint DIM, parity, and milk FTIR spectra modeling did not improve the prediction of pregnancy status as compared to the model with milk FTIR spectra alone. The inclusion of herd and year only slightly improved predictions. Here, the herd effect was the most influential of all the on-farm predictors. The herd effect may account for differences in feeding systems and management between dairy herds. Nevertheless, as practical applications often require predictions across dairy farms [12], herd CV was also used to evaluate a more realistic scenario in routine evaluations. In this case, on-farm information such as herd, DIM, and parity may be excluded from the prediction model.

The inclusion of whole-genome and/or top markers with large effects provided greater predictions for both the kernel methods and BayesB, especially for $\kappa \text {-}$CN and $\beta \text {-}$CN. Genotype data increased $\text {R}^2$ possibly because of the high heritability of these traits. Pegolo et al. [22] reported genomic heritability estimates of 0.83 and 0.68 for $\kappa \text {-}$CN and $\beta \text {-}$CN, respectively. These estimates were higher than those for other milk composition traits, ranging from 0.13 to 0.66. Our kernel methods showed that $\text{R}^2$ was higher for some traits when the top three markers were incorporated. Hence, these quantitative trait loci have a strong influence. Pegolo et al. [22] identified significant SNPs on chromosomes 6 and 11 for milk protein fractions. Within the two CV, we found the same markers as in Pegolo et al. [22] for $\kappa \text {-}$CN and $\beta \text {-}$CN using multiple kernel learning, while multilayer BayesB further identified common markers associated with TWP, $\alpha \text {-}$LA, $\alpha _{S1}\text {-}$CN, and $\alpha _{S2}\text {-}$CN. The observed improvement in predictive performance of BayesB for all traits may have been the result of effectively distinguishing SNPs or spectra with large effects from those with small effects. Recent studies reported that including genomic information influences prediction positively. Wang and Bovenhuis [19] obtained comparatively better predictions for milk fat component traits when they combined milk FTIR data and three polymorphisms of the diacylglycerol acyltransferase 1 (DGAT1) K232A, stearoyl-CoA desaturase 1 (SCD1) A293V, and fatty acid synthase (FASN) genes. Ho et al. [11] showed that the addition of genomic signals such as principal components calculated from a genomic relationship matrix and fertility genomic estimated breeding values to spectral data enhances the prediction of conception at first insemination. Therefore, integrating genotype information or including markers with large effects with milk FTIR spectra might improve the prediction of certain traits.

There may be a limited number of females with genotype information, as most of them are genotyped for genomic selection purposes only. For this reason, we investigated the combination of pedigree data with milk FTIR spectral information via a numerator relationship matrix. This approach yielded $\text{R}^2$ similar to or lower than those obtained from the genomic relationship matrix. Nevertheless, for some traits such as TWP, $\kappa \text {-}$CN, and $\alpha _{S1}\text {-}$CN, the predictive performance including pedigree information was slightly better (higher $\text{R}^2$) than that obtained using milk FTIR or on-farm data alone. Therefore, leveraging pedigree information is a feasible alternative when genotype information is not available.

### Comparison of multiple kernel learning and multilayer BayesB to PLS

We compared kernel methods, BayesB, and PLS in terms of their effectiveness in including heterogeneous information for the phenotypic prediction of different milk protein component traits. The model with milk FTIR spectra alone served as the reference baseline. For both CV scenarios, the kernel methods and BayesB had similar predictive performance compared to PLS using only spectral information for non-fraction traits. However, BayesB delivered relatively better results for many of the fraction traits. Compared to multiple kernel learning and PLS, multilayer BayesB also showed better predictions when either on-farm data or both on-farm data and genomic information plus milk FTIR spectra were used. Ferragina et al. [5] reported superior predictive performance for BayesB compared with Bayesian ridge regression, BayesA, and PLS when milk FTIR spectral information was used. Bonfatti et al. [29] reported that $\text{R}^2$ for milk protein fractions (g/L of milk) such as TWP, $\beta \text {-}$CN, and $\alpha _{S1}\text {-}$CN derived from BayesB and BayesC, using spectral data alone, showed slightly better prediction than that of PLS. However, the differences were small. Our results generally corroborated their findings and further demonstrated that multilayer BayesB can be a useful tool to integrate heterogeneous data. This discovery is consistent with recent studies in which multilayer Bayesian regression models were applied to integrate genomics, transcriptomics, or methylation data [30, 31]. As described earlier, BayesB is a variable selection method that distinguishes predictors with large effects from those with small effects. Numerous FTIR spectrum regions may have marginal effects on a target trait [5]. Thus, BayesB can effectively identify the wavenumber ranges with large effects on the traits of interest.

To the best of our knowledge, this is the first study to apply multiple kernel learning for phenotypic prediction based on the construction of a spectral relationship matrix among individuals according to their milk FTIR spectral profiles. The advantage of kernel methods is that they can accommodate multiple sources of information provided that the kernels can be constructed from each information set [23]. An important example is a genomic relationship matrix embedding the genomic profiles of individuals. However, the construction of kernels does not preclude using non-genomic sources. For instance, Hu et al. [32] developed a relationship matrix among *Arabidopsis thaliana* lines based on their methylation profiles to conduct methylation-based phenotypic prediction. Krause et al. [33] used a hyperspectral reflectance relationship matrix from hyperspectral bands in wheat to predict grain yield. Li et al. [34] applied a transcriptomic-based relationship matrix among *Drosophila melanogaster* lines using tiling arrays to predict nine traits including startle responses. In our study, kernel methods were extended to accommodate milk FTIR spectral data. Overall, kernel methods showed a lower predictive performance than that of BayesB. This might be attributed to the fact that kernel methods assume a common wavenumber variance whereas BayesB performs variable selection. Therefore, using a weighted spectral relationship matrix by putting a prior weight to each individual wavenumber (if available) may enhance prediction. However, kernel methods offer a straightforward avenue to integrate additive genetic effects based on pedigree (i.e., numerator relationship matrix).

When genomic or pedigree information was added, the prediction $\text{R}^2$ values for PLS decreased for numerous traits. It is likely that this occurred because the standard PLS widely used in spectra analysis does not clearly differentiate between contributions from genomics/pedigree and milk FTIR spectra. We also explored predictive performance using the pre-treatment data because using derivative milk spectra or removing noisy spectra regions may improve prediction performance [3]. In fact, the use of informative spectra led to an increase in $\text{R}^2$ values for all the traits compared to the use of non-treated spectra, however, prediction performance did not improve greatly when genomic or pedigree data were included. As stated earlier, multiple kernel learning and multilayer BayesB can better accommodate multiple heterogeneous data than PLS for a prediction purpose. The PLS method maximizes variance conditional on the response variable regardless of the source of predictors. Further studies are warranted to improve the predictive ability of PLS in the context of data integration.

## Conclusions

This study investigated the effectiveness of kernel methods, BayesB, and PLS at integrating heterogeneous data including milk FTIR spectral, on-farm, genomic, and pedigree data for predicting milk protein traits. Multiple kernel learning and multilayer BayesB can potentially improve milk protein trait prediction performance by correctly assigning different weights or priors for genetic (genomic or pedigree) and milk FTIR spectral components. In particular, multilayer BayesB was identified as the best predictive model. The present study provides alternative statistical methods for spectra-based predictions.

## Data Availability

The data analyzed are available from the corresponding author on reasonable request.
